# A decision support system based on recurrent neural networks to predict medication dosage for patients with Parkinson's disease

**DOI:** 10.1038/s41598-024-59179-0

**Published:** 2024-04-10

**Authors:** Atiye Riasi, Mehdi Delrobaei, Mehri Salari

**Affiliations:** 1 Department of Biomedical Engineering, Faculty of Electrical Engineering, K. N. Toosi University of Technology, Tehran, Iran; 2 Department of Mechatronics, Faculty of Electrical Engineering, K. N. Toosi University of Technology, Tehran, Iran; 3 Department of Electrical and Computer Engineering, Western University, London, ON Canada; 4 Department of Neurology, Shahid Beheshti University of Medical Sciences, Tehran, Iran

**Keywords:** Biomechatronic systems, Artificial intelligence, Deep learning, Remote monitoring, Pharmacological management, Sequential learning, Long short-term memory, Data mining, Data processing, Machine learning

## Abstract

Using deep learning has demonstrated significant potential in making informed decisions based on clinical evidence. In this study, we deal with optimizing medication and quantitatively present the role of deep learning in predicting the medication dosage for patients with Parkinson's disease (PD). The proposed method is based on recurrent neural networks (RNNs) and tries to predict the dosage of five critical medication types for PD, including levodopa, dopamine agonists, monoamine oxidase-B inhibitors, catechol-O-methyltransferase inhibitors, and amantadine. Recurrent neural networks have memory blocks that retain crucial information from previous patient visits. This feature is helpful for patients with PD, as the neurologist can refer to the patient's previous state and the prescribed medication to make informed decisions. We employed data from the Parkinson's Progression Markers Initiative. The dataset included information on the Unified Parkinson's Disease Rating Scale, Activities of Daily Living, Hoehn and Yahr scale, demographic details, and medication use logs for each patient. We evaluated several models, such as multi-layer perceptron (MLP), Simple-RNN, long short-term memory (LSTM), and gated recurrent units (GRU). Our analysis found that recurrent neural networks (LSTM and GRU) performed the best. More specifically, when using LSTM, we were able to predict levodopa and dopamine agonist dosage with a mean squared error of 0.009 and 0.003, mean absolute error of 0.062 and 0.030, root mean square error of 0.099 and 0.053, and R-squared of 0.514 and 0.711, respectively.

## Introduction

Parkinson's disease (PD) is a neurodegenerative disease caused by dopamine-producing cell death in the brain. The loss of dopaminergic neurons in the substantia nigra leads to depletion of dopamine in Parkinson's disease. According to a 2022 study by the Parkinson's Foundation, almost 90,000 people in the U.S. are diagnosed with PD each year. That is a significant increase of 50% compared to the previous estimate of 60,000 cases diagnosed each year^1^. PD can cause severe disability and lead to a wide range of motor and non-motor symptoms. Motor symptoms include tremors, rigidity, bradykinesia, and gait disorders, and featured non-motor symptoms are cognitive impairments, constipation, depression, hallucination, anxiety, and sleep disturbance^2^. There is no guaranteed cure for PD. However, ongoing disease management, which generally includes medication, can improve patients' quality of life^3,4^. In some instances, an invasive operation called deep brain stimulation may be recommended^5^.

The main types of medication for people with PD include levodopa (LD), dopamine agonists (DA), monoamine oxidase-B inhibitors (MAOB-I), catechol-O-methyltransferase inhibitors (COMT-I), amantadine, and anticholinergics^6^. This medication therapy can be converted into levodopa equivalent daily dosage (LEDD) suitable for continuous injection pumps such as Duopa. Duopa is a suspension consisting of carbidopa and levodopa. It is infused during a constant 16 h of pump work based on levodopa equivalent dosage of previous oral medications prescribed by the neurologists. It also allows an extra scheduled suspension dosage if the off-time symptoms increase^7^.

Levodopa is a proven treatment for improving motor symptoms in patients with PD when taken in appropriate dosages^4,8–10^. Over time, the prescribed or equivalent levodopa dosage may need to be increased for optimal long-term results^11^. Furthermore, patients with an increased dose of levodopa are at risk of levodopa-induced dyskinesia^12–14^. Artificial intelligence (AI) can effectively provide a medical model based on the biological profile of each patient with a complex combination of symptoms and suggest an optimum treatment to physicians. Such a model can be used in a Duopa pump for remote management of PD or even in a mobile application to support patients in situations where they do not have access to their physicians. Nowadays, for several diseases, due to the importance of long-term management of the disease and the variety of symptoms between patients, and even in the case of the same patient at different times, the existence of a decision support system (DSS) is a critical issue^15,16^. For individuals with PD, a personalized strategy to calculate the appropriate LEDD for each patient could be highly beneficial in suggesting the optimal dosage. However, the physician should ultimately adjust the recommended dosage for optimal results.

## Literature review

Specific guidelines recommend medication for treating PD^17,18^. Still, various symptoms and long disease duration encourage researchers to develop decision-support algorithms based on the trajectory of consecutive patient visits. Timotijevic et al.^19^ successfully presented the benefits of mobile clinical decision support systems (CDSS) and showed that the user needed different CDSS despite various theoretical guidelines. For instance, algorithm-based CDSS may be implemented to support collaborative decision-making between clinicians, patients, and caregivers, to increase the flexibility of diagnostics among clinicians, and to gather information from different sources. The EU 2020 project PD-Manager has created a framework for developing a DSS^20^. This framework aimed to develop and assess a mobile health (m-health) platform for managing PD. The PD-manager DSS suggests the treating neurologist, who calibrates them and makes the final decision.

Bohanec et al.^21^ applied data mining and expert rules to the DEX decision expert method using DEXi software^22^. DEX method is a multicriteria qualitative approach with a hierarchical structure in which lower-level attributes are related to higher ones by decision rules^23^. Using DEXi, they acquired expert knowledge, and after subsequent analysis, they found situations in which patients need to change their medication in three levels: Yes, Maybe, or No. They analyzed various symptoms and epidemiological data of patients with PD, including motor symptoms such as bradykinesia, tremor, gait problems, dyskinesia, and on/off fluctuations, as well as non-motor symptoms such as daytime sleepiness, cognitive disorders, impulsivity, depression, and hallucinations. They also took into account factors such as the age of the patients, their employment status, the duration of their illness, and whether they lived alone or with a caregiver. The severity of symptoms was classified into three groups: problematic, maybe, and normal.

Boshkoska et al.^24^ in 2020 improved the previous work by detecting not only changing situations but responding to how to change the therapy. They developed a state transition model to show the current and later medication; data-mined and expert-defined rules compile the transitions between states. They considered various forms of any possible LD, DA, and MAOB-i combinations. In addition, the transitions in the proposed model included decreasing/increasing the medication dosage, including/excluding medication, and changing the medication type from current to new. The model was evaluated using the Parkinson's Progression Markers Initiative (PPMI) dataset and a questionnaire answered by seventeen neurologists from four European countries.

Their model's input attribute focused on motor symptoms such as dyskinesia (off duration and intensity), tremor (rest, action, postural), rigidity and bradykinesia, mental problems including impulsivity, cognition, hallucination and paranoia, epidemiologic data on patient’s age and activity of daily life (ADL) and comorbidities like cardiovascular problems, hypertension, and low blood pressure. In this study, the severity of symptoms was accounted for in the binary form of yes/no. Based on Unified Parkinson's Disease Rating Scale (UPDRS) scores, only a score of 4 is considered a yes for paranoia. Patients are under 65 years old, 65–75 years old, and over 75 years old. Patients whose percentage of activity was below 60 were considered inactive, and those over 60 were active. For motor symptoms, hallucinations, and all other symptoms, scores of 0–1 and 2–4 are considered as no and yes, respectively.

In 2021, Kim et al*.*^25^ suggested a different model based on reinforcement learning (RL) and provided suggestions on the optimal combination of medication. It was claimed that they were the pioneers in utilizing artificial intelligence for medication recommendations. They used the Markov decision process^26^ to demonstrate the sequence of visits for each patient. The proposed model by Kim consists of disease states at the current visit, actions including a combination of three groups of medication (LD, DA, and other PD medication), and finally, rewards, which are the patient’s response to medication. The model aims to minimize the sum of total UPDRS scores. A virtual agent in a given state selects an action through a trial and error process from many actions to maximize future rewards. The effect of the medication on each patient was assessed by UPDRS Part III in the ON condition of the medication using PPMI data. AI has also been used to treat diseases like sepsis^27^, epilepsy^28^. However, this study was the first attempt to apply it to manage PD medication.

In other research, Baucum et al*.*^29^ extended the use of RL to propose a patient-specific medication regimen through wearable sensors in 2023. They used a small data set of 26 patients with PD who were monitored with wrist-mounted sensors for two six-day periods. Their model includes bradykinesia and dyskinesia scores extracted from wrist movement tracker sensors, time from the last dosage, and three types of only levodopa dosage (Immediate release, controlled released, and Rytary which is an extended-release of levodopa) and total equivalent dosage as states, and 22 actions from no medication to a maximum of two-drug levodopa combination from different dosages. They also showed that although their wearable-based RL policies are tested on a small amount of real data, they can outperform PPMI-based policies.

None of the research studies on PD utilized deep learning time series methods to predict personalized information based on sequential patient visits. However, recently, some studies that employed comparable methods to classify the stage of Alzheimer's disease (AD) showed the potential of time series in managing long-term disease based on the visit history of the patients^30,31^. Using recurrent neural networks (RNNs), Pan et al.^31^ investigated the progression of AD to classify the severity of the disease. They applied neuropsychological test results, genetics, and demographics data of patients in the first, sixth, and twelfth months as input features of the model. They considered a three-dimensional output layer including normal, mild cognitive impairment, and AD. Their model shows that the relation between symptoms in previous consequence visits can help more accurate predictions for the next stage^30^.

### Objectives

After conducting extensive research, we found that none of the existing models fully address the issue of determining the appropriate medication dosage for patients with PD. Therefore, this study proposes a solution by offering a method that predicts personalized LEDD of 5 main medications based on previous dosages and new measurements of patients' motor and non-motor symptoms. We believe the suggested method's reported performance is a promising foundation for other researchers who wish to assist clinicians and patients in managing oral intake or injection of PD medication. These findings could also be used for remote monitoring and management of patients with PD. However, due to the high sensitivity of medical systems, it is essential to adjust expected values hierarchically under the supervision of a neurologist.

## Materials and methods

### Data description and visualization

The dataset used in this study was extracted from the PPMI database^32^, a comprehensive, prospective, computational study that includes imaging data, clinical tests, biospecimens, and genetic biomarkers to track the progression of PD. Table 1 shows the contributed files of PPMI used in the current study. The PPMI has been collected at various centers, including the United States, Europe, Israel, and Australia. This diversity prevented the data collection process from being limited to a single hospital or clinic setting or the availability of some medications in a specific location.

**Table 1:** Informative files from the PPMI database contributed to this study.

Folder name in PPMI database	Description
Subject_Characteristics	Age at visits, gender
Motor MDS-UPDRS	MDS-UPDRS (I, II, III, IV), modified Schwab England ADL
Medical_History	LEDD_Concomitant_Medication_Log
Hoehn & Yahr stage	Hoehn and Yahr scale

After merging severity scores and medication records based on visit dates, missing values were removed from the data set (0.6% of all visits). Furthermore, patients’ data for those with medication dosages higher than 2500 mg were considered outliers and removed from the study.

Types of PD-related medication were categorized into five main groups: LD, DA, MAOB-i, COMT-i, and Amantadine. Table 2 shows the repetitive brands of each group in the database.

**Table 2:** Repetitive types of five main groups of medication in the database.

Medication group	Repetitive medication
LD	Sinemet, Parcopa, Rytary, Madopar and Carbidopa Levodopa
DA	Mirapex, Requip, Neupro and Apokin
MAOBi	Selegiline, Rasagiline and Safinamide
COMTi	Tolcapone, Entacapone
Amantadine	Amantadine, Gocovri, Osmolex andApomorphine

All reported medications were reviewed to be consistent across the log. Spelling mistakes were corrected, and different representations of medication names were considered the same. For example, “LEVODOP,” “LEVODAP,” and “L-DOPA” were replaced by levodopa. The mean value was considered for cases where the same medication regimen was used simultaneously with variable doses^33^.

**Figure 1:**
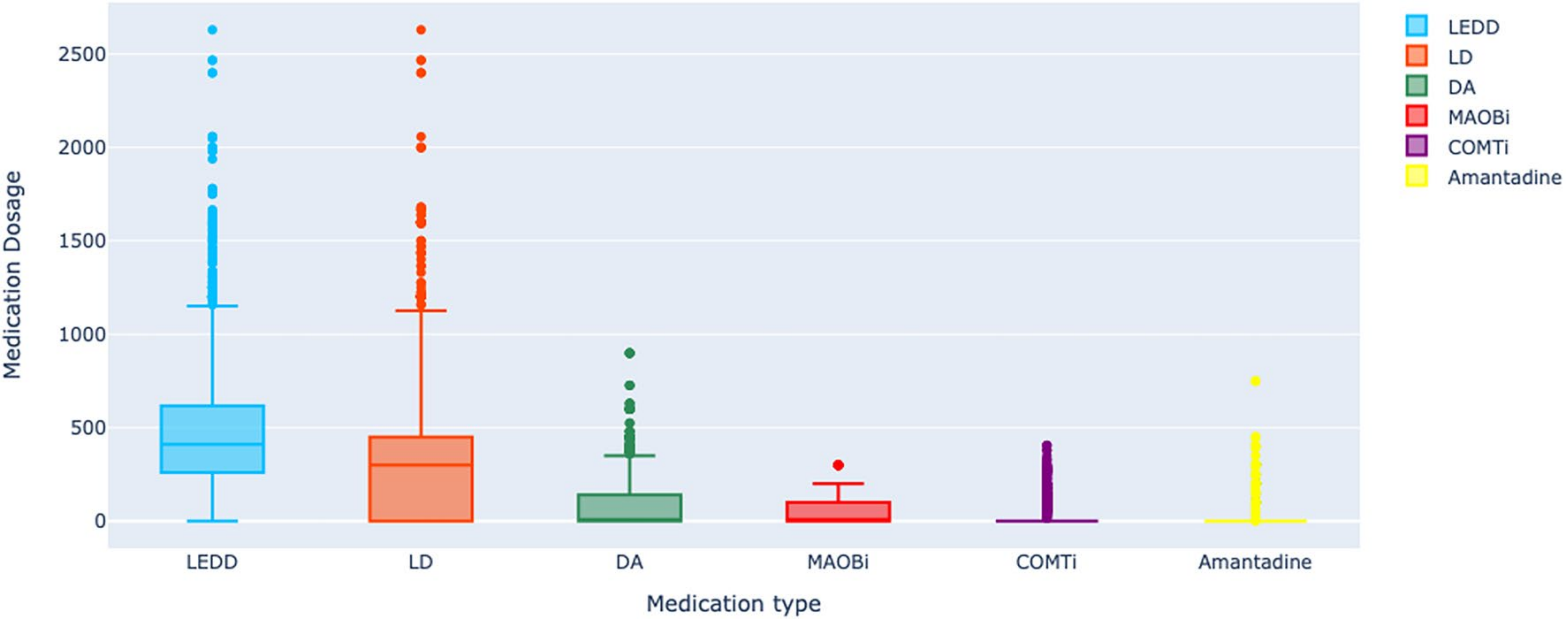
Box plots of medication dosage (mg) used in different visits of all patients for five main groups of PD medications including LD, DA, MAOB-i, COMT-I, and amantadine. LEDD is the sum of 5 medications. The medication dosage in the y-axis is based on the levodopa equivalent dosage.

After medication preprocessing and categorizing them into five main groups, The final data set's medication usage is displayed in Fig. 1 through box plots.

The severity scores of the symptoms were only considered in the “ON state.” This study analyzed a dataset comprising 4,143 patient visits for PD, including 1,614 visits from female patients. All sub-items of the Movement Disorder Society‐sponsored revision of the UPDRS (MDS‐UPDRS)^34^ in ON medication states, Activities of Daily Living, age, Hoehn and Yahr scale were included in this study. We chose not to exclude any features based on feature selection methods to ensure greater generality. Instead, we calculated the sum of certain items, such as the sum of a specific item on both the left and right sides of the body.

Table 3 summarizes the rating scores of the patients, including range, mean, and standard deviation for all visits.

**Table 3:** Summary of the rating scores of the patients.

Patient’s measurement	Scores range	Mean (std)
UPDRS-Part1	[0,24]	6.82 (4.09)
UPDRS-Part2	[0,45]	9.61 (6.62)
UPDRS-Part3	[0,89]	22.50 (12.33)
UPDRS-Part4	[0,17]	1.95 (2.85)
Total UPDRS	[2,151]	39.4 (18.80)
Activities of daily living (%)	[10,100]	86.38 (11.38)
Age at visit	[29,91]	64.59 (9.74)
Hoehn and Yahr	[1,5]	1.9 (0.56)

**Figure 2:**
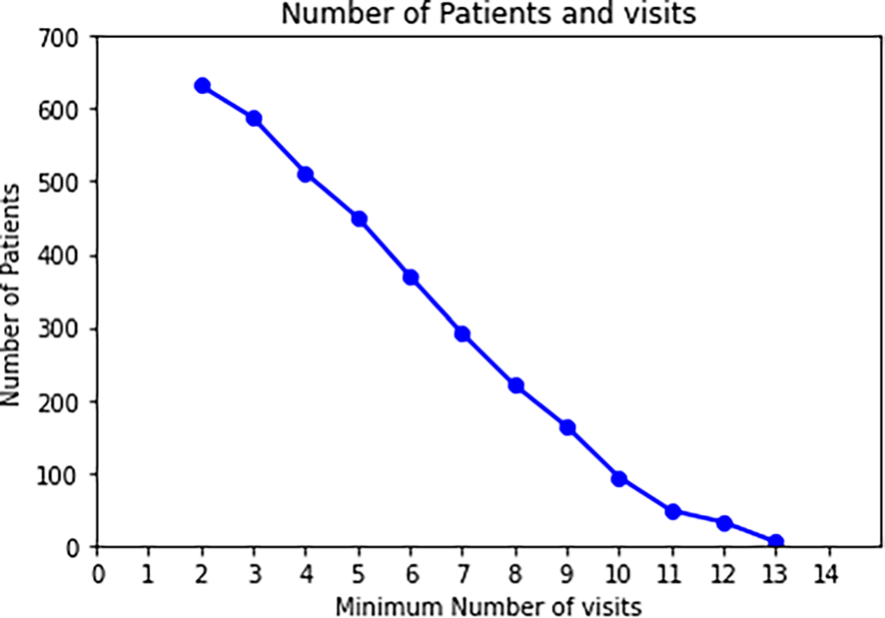
Representation of the number of patients and their corresponding minimum number of visits. Among the 673 patients, 632 had a minimum of two consecutive visits. Patients without follow-up visits (i.e., with only one visit) were dropped from the study and not shown on the plot.

Figure 2 displays the total count of patients with their minimum number of visits. As can be seen, out of the 673 patients, 632 (260 identified as female) had a baseline visit and were followed up on at least one occasion.

### Proposed framework

The proposed model uses RNNs to provide a sequence-by-sequence decision support framework based on patients' subsequent visits. A multi-layer perceptron (MLP) network was also applied to compare the results with a non-sequential method. This approach considers every patient with a visitation history as an input sample. Considering the sequential time series issue in deep learning to apply crucial attributes of past visits makes the model more reliable and individualized than predicting dosage based on a single point in time^35^.

**Figure 3:**
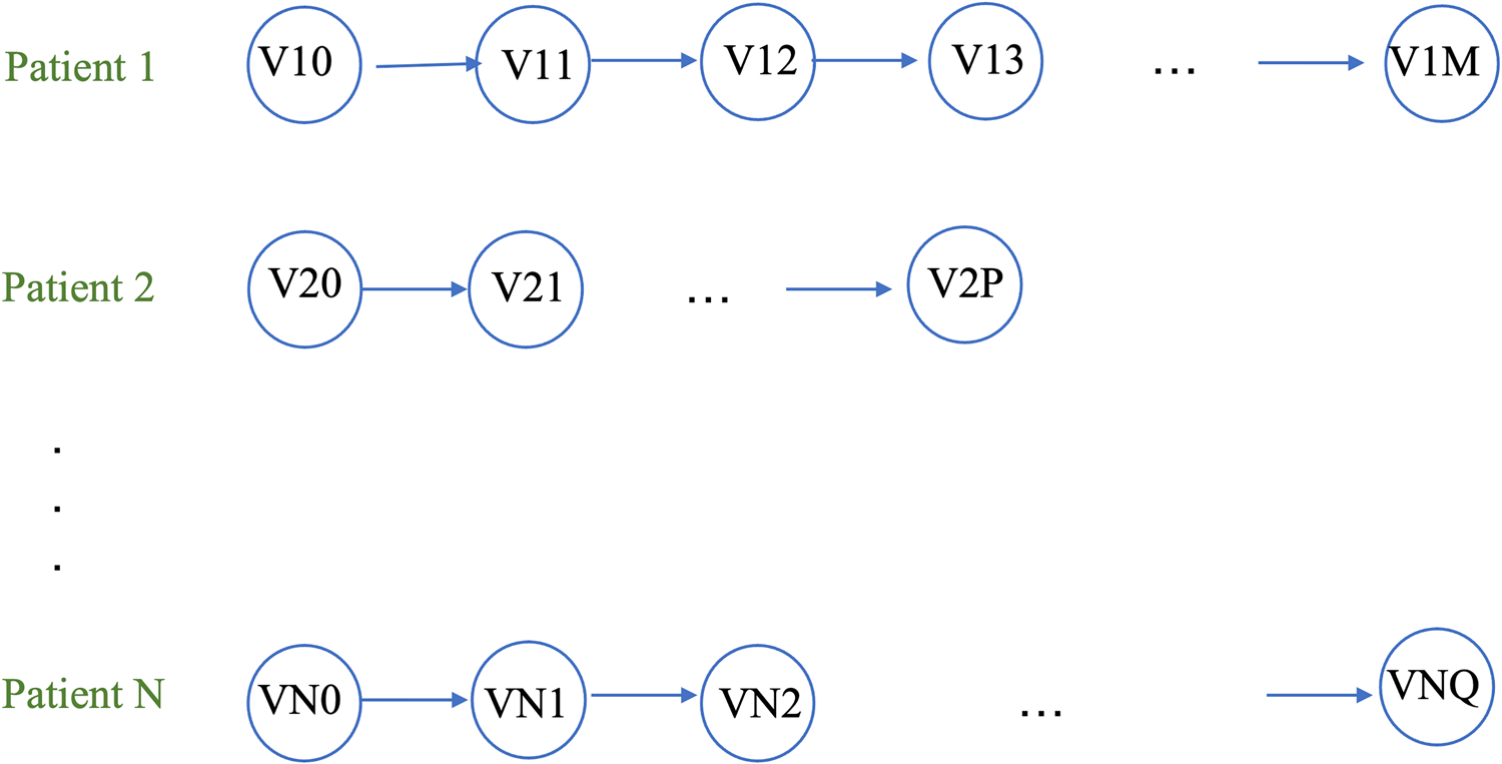
The raw data format; where subject N is the Nth patient, Vij indicates the jth visit for the ith patient with different number of visits for each patient (M#P#Q).

**Figure 4:**
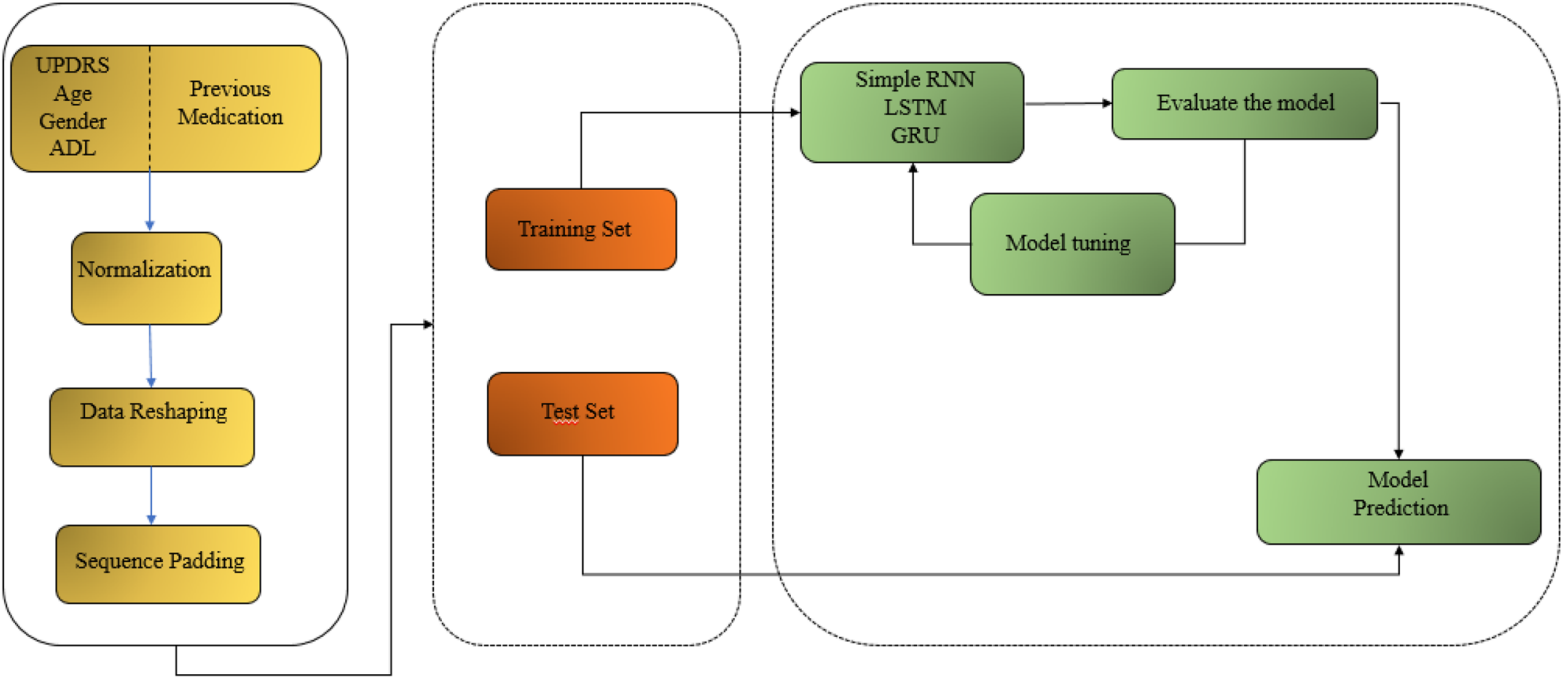
Decision support framework using sequential data and recurrent neural networks. This framework consists of three main sections of 3D data preparation with a lag of five visits history, splitting data into training and test sets, and finally tuning the model with train data and making a prediction using the test set.

Figure 3 displays multiple visits for each patient, while only the last five visits were considered for all patients. Figure 4 provides an overall framework for the proposed DSS. The steps in this framework are as follows: (1) data preparation to manage outliers and missing values, (2) data normalization, (3) data reshaping, (4) sequence padding, (5) recurrent models fitting on the training set, and (6) models evaluation based on the test set.

### Recurrent neural network models to predict medication dosages

The deep learning regression maps the non-linear relation between input characteristics and outputs. This study aimed to show the ability of sequence-by-sequence deep learning regression models, including simple recurrent neural network (Simple-RNN)^36^, long short-term memory (LSTM)^37^, and gated recurrent units (GRU)^38^ to solve the proposed regression problem and suggest the next equivalent medicine dosage based on each patient's consecutive visits. The RNNs, acting like a chain, allow the model to predict the medication dosage based on previous time-step computations. Non-sequential data was entered into a base MLP model (with no recurrent connections) of the same structure of recurrent networks for comparing the results.

The input data for the models are customized based on the different visits of each individual by keeping the order of the reference visit to the last recorded visit of each patient. The patients with only one recorded visit were removed from the experiment. Because of the different number of visits for patients, according to Fig. 4, the last five visits were considered for all patients. The input time delay was padded to five for those with less than five visits by considering zeros for features.

The models were constructed using open-source Python software that utilized the Tensorflow and Keras libraries. For all models, 90% of the data were selected to train and 10% for the test, and the regularisation technique L2 was used for models to avoid overfitting. For time-series recurrent neural networks, 3D input data of 632 patients (568 for training and 64 for testing) with five time-step visitation histories was fed into the network as input data. However, In our MLP network, each visit was considered an independent input sample without considering the visitation history of patients, and 4143 input samples (3728 for train and 415 for testing), with the same features as RNNs (UPDRS, age, gender, ADL and previous visit medication) were fed into the network. Table 4 shows the architecture and best parameters of RNNs obtained by trial and error. In the output layer, the Selu activation function is used. Selu can self-normalize the output of neural networks, improving training stability and helping networks converge faster because of its internal normalization^39^.

**Table 4:** Architecture and parameters of proposed sequence-by-sequence model.

Layers	Type
Input	Sequential-3D array (–,5,61)
Hidden 1	SimpleRNN/LSTM/GRU, 50 units, activation = relu, kernel regularization = l2
Dropout 1	20%
Hidden 2	SimpleRNN/LSTM/GRU, 50 units, activation = relu
Dropout 2	20%
Dense	Dense, activation = selu
Regression output	Optimizer = adam, loss function = mean squared error

### Performance evaluation

The proposed framework was evaluated using four statistical performance measures, including R-squared (R^2^), mean squared error (MSE), mean absolute error (MAE), and root mean squared error (RMSE).

In all the following evaluation metrics, n is the total number of test samples, ${y}_{i}$ is the observed output of ith sample, $\widehat{{y}_{i}}$ is the corresponding predicted value, and $\overline{y }$ represents the mean of the observed values.

The R^2^ score, also known as the coefficient of determination, can be expressed as

1 $${R}^{2}=1-\frac{\sum_{i=1}^{n}{({y}_{i}-\widehat{{y}_{i}})}^{2}}{\sum_{i=1}^{n}{({y}_{i}-\overline{y })}^{2}}$$

The R^2^ score represents how well the model’s dependent variable traces the variability of independent input features. A fit model can achieve an R^2^ score of 1. In contrast, a random model's R^2^ score is close to 0.

The mean squared error can be expressed as

2 $$MSE=\frac{\sum_{i=1}^{n}{({y}_{i}-\widehat{{y}_{i}})}^{2}}{n}$$

that represents how much the predicted value is close to the observed value in a regression model.

The mean absolute error (MAE) is the average of differences between observed and predicted values and is given by

3 $$MAE=\frac{1}{n}\sum_{i=1}^{n}\left|{y}_{i}-\widehat{{y}_{i}}\right|$$

Finally, the root mean squared error shows the deviation between predictions and observed values using Euclidean distance and can be calculated by

4 $$RMSE=\sqrt{\frac{\sum_{i=1}^{n}{({y}_{i}-\widehat{{y}_{i}})}^{2}}{n}}$$

## Results

The effectiveness of the proposed medication dosage prediction system, utilizing both recurrent neural networks and simple MLP, was assessed using R^2^, MSE, MAE, and RMSE metrics. Table 5 displays the outcomes of the system evaluated on the Levodopa Equivalent dosage prediction for the five primary groups of medication and the total LEDD. The MLP model has a weak R^2^ score of zero compared to the recurrent neural networks. Mostly, it yields negative R^2^ scores, indicating that the model cannot keep track of input feature changes, and the output results are based on a constant average. The LSTM and GRU recurrent neural networks outperform Simple-RNN for the main medication types: LD, DA, and MAOBi. The bolded outcomes highlight the best model for each medication type.

**Table 5:** Evaluation results of prediction using MLP, Rnn, LSTM, and GRU.

Medication	Model	MSE	MAE	RMSE	R^2^ score
LD	MLP	0.014	0.080	0.117	0.000
	RNN	0.012	0.074	0.110	0.380
	**LSTM**	**0.009**	**0.062**	**0.099**	**0.514**
	GRU	0.013	0.069	0.115	0.334
DA	MLP	0.016	0.098	0.125	0.000
	RNN	0.004	0.038	0.063	0.586
	**LSTM**	**0.003**	**0.030**	**0.053**	**0.711**
	GRU	0.003	0.034	0.057	0.657
MAOBi	MLP	0.026	0.160	0.162	-0.013
	RNN	0.008	0.062	0.092	0.622
	LSTM	0.007	0.055	0.083	0.691
	**GRU**	**0.006**	**0.050**	**0.078**	**0.727**
COMTi	MLP	0.010	0.042	0.102	-0.003
	RNN	0.009	0.038	0.097	0.489
	**LSTM**	**0.008**	**0.031**	**0.092**	**0.542**
	GRU	0.011	0.038	0.103	0.427
Amantadine	MLP	0.012	0.069	0.108	-0.002
	**RNN**	**0.007**	**0.037**	**0.083**	**0.390**
	LSTM	0.010	0.056	0.104	0.032
	GRU	0.009	0.044	0.095	0.204
LEDD	MLP	0.015	0.089	0.123	0.000
	RNN	0.014	0.079	0.120	0.362
	**LSTM**	**0.009**	**0.063**	**0.095**	**0.598**
	GRU	0.013	0.076	0.115	0.417

The most suitable model.

**Figure 5:**
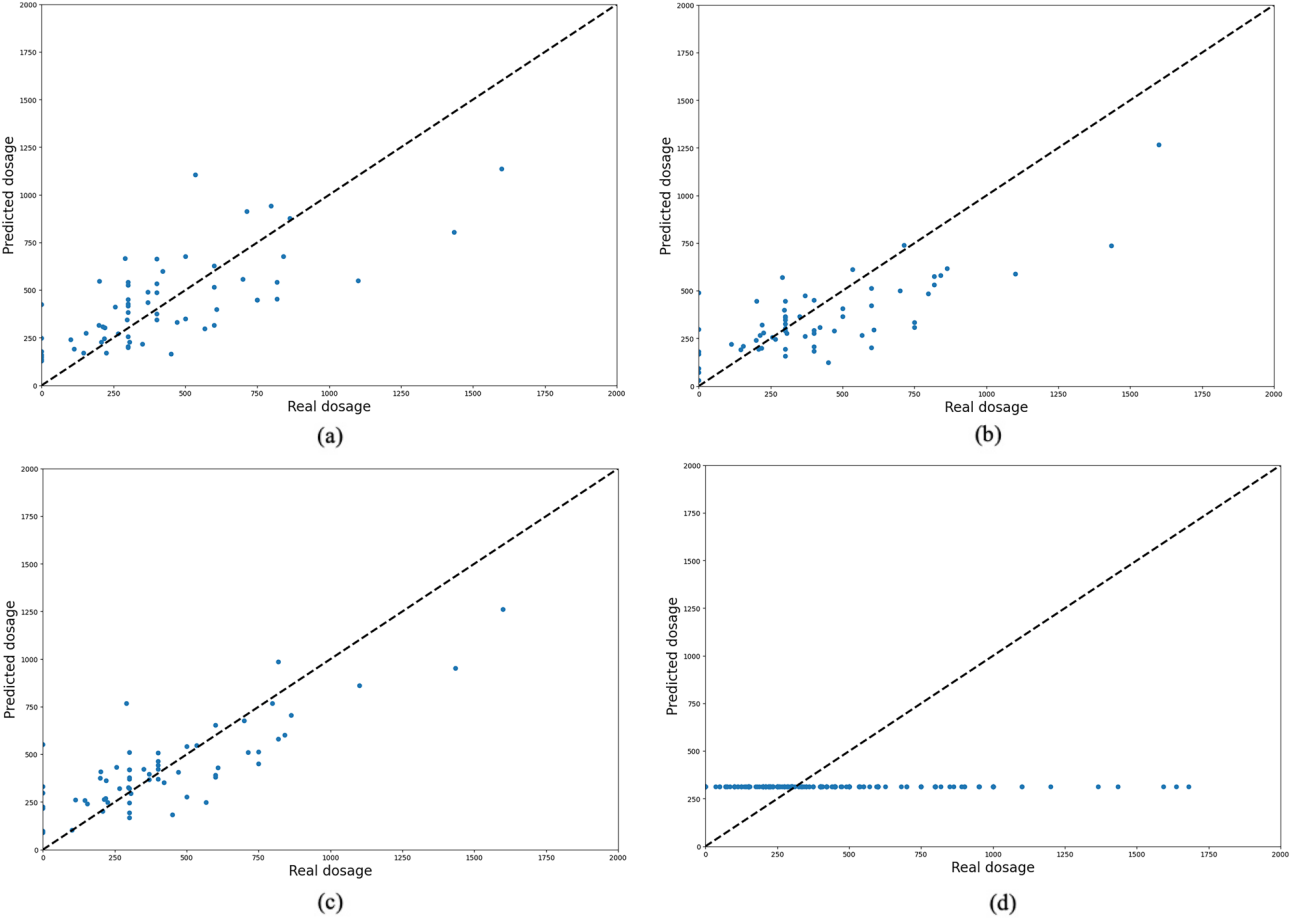
Prediction results of models for LD medication dosage using (**a**) RNN, (**b**) LSTM, (**c**) GRU, and (**d**) MLP models. The x-axis is the real dosage, and the y-axis is the predicted dosage for LEDD. In RNNs, most of the points are close to the 45-degree line, which means good prediction results; however, in the MLP model, the output is constant and near the average of all visits’ dosages.

**Figure 6:**
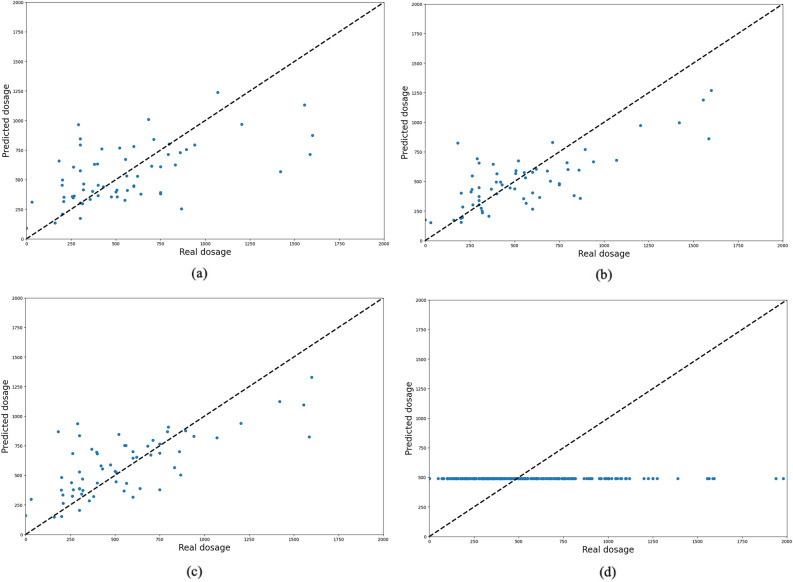
Prediction results of models for LEDD medication dosage using (**a**) RNN, (**b**) LSTM, (**c**) GRU, and (**d**) MLP models. The x-axis is the real dosage, and the y-axis is the predicted dosage for LEDD. In RNNs, most of the points are close to the 45-degree line, which means good prediction results; however, in the MLP model, the output is constant and near the average of all visits’ dosages.

Figures 5 and 6 indicate the predicted and observed values of the test samples for LD and LEDD medication using four models. As can be seen, sequential time series models have the potential to predict the dosage of medication based on previous visits’ history. However, with non-sequential models (i.e., MLP), the prediction will be approximately around an average of whole samples, and the input features of each patient will not affect the prediction results.

## Discussion

This study aimed to show the ability of time series sequence by sequence recurrent neural network regression algorithms to predict the medication dosage of PD patients with consecutive visit history. Since managing PD requires long-term care and every patient experiences unique symptoms over time, having a decision support system to suggest the best dosage for each patient could be a valuable accomplishment. Such a system can aid primary care physicians when they cannot access a neurologist. Additionally, as telemedicine technology improves, decision support systems can help manage diseases remotely under the guidance of a specialist by computing the proper dosages of medication based on the information from wearable sensors. At the next level, the recommended dose could be remotely administered using a smart infusion pump or displayed on patients’ smartphones to take the medication orally.

Among the wide variety of information and documents registered in the records of patients with PD, we selected limited features as input to the system with the consultation of some experienced neurologists and previous studies^24,25^, which have the most significant impact on the prescribed medication dosage. This enhances the system's simplicity and speed. Our selected input features include all motor and non-motor sub-items of the UPDRS in ON medication states, activities of daily living, the Hoehn and Yahr scale, demographic details, and previously used medication dosages. In order to reduce the dimensions of the input set, the features from the same type were fed into the network as the sum of all its subitems. For example, the tremor score was considered the summation of postural, action, and rest tremor, and some other features for the right and left side of the body were added together.

Furthermore, extensive preprocessing was needed to prepare the medication log. The medication log comprised a range of medication usage for each patient in different periods. We matched the periods of medication usage in the log with the date of visits for each patient. When the same medication was recorded with different dosages, we took the mean dosage^33^. Various brand names were also recorded in the medication log; hence, we categorized all the medications into five main groups: LD (i.e., Sinemet, Parcopa, Rytary, Madopar), DA (i.e., Mirapex, Requip, Neupro, Apokyn), MAOB-i (i.e., Selegiline, Rasagiline, Safinamide), COMT-i (i.e., Tolcapone, Entacapone), and Amantadine. The daily dosage and the frequency for all medications were recorded, and using conversion factors developed by Tomlinson et al.^40^, the daily dosage of levodopa equivalent was calculated and recorded. The sum of all levodopa equivalent dosages at a specific time point formed the LEDD for that time point.

Several studies have aimed to create decision-support systems for patients with PD. Initially, most systems employed traditional decision trees and DEX methods to suggest the most effective medication combination. However, in 2021, researchers attempted to use reinforcement learning to determine the optimal medication combination^25^.

In the case of other diseases with long time management, it was shown that the information from previous visits could improve the accuracy of Alzheimer's disease stage prediction using LSTM classification^30,31^. The similarity of AD and PD in long-term management with consecutive visits led us to apply RNNs on PD in the case of medication dosage suggestions. To our knowledge, our study is the first attempt to suggest the quantitative dosage of medications using artificial intelligence. Another advantage of this research is considering the five main types of medication and their dosage. In comparison, previous works only consider two or three types without mentioning a dosage value for each type^21,24,25^. We showed the applicability of regression sequence by sequence time series models for predicting the dosage of five main types of PD medication from new measurements using measurements and prescribed medications from previous visits.

We took several measures to ensure the reliability of our model. We selected a diverse dataset from various world centers and processed it precisely to obtain real and relevant data from different regions without any estimation. We also used the L2 regularization kernel to prevent overfitting, making predicting unseen data more accurate. Finally, we repeated algorithms with different hyperparameters to obtain proper results. However, some methods to guarantee reliability were left unimplemented. Adding noise to the dataset can improve the framework's resilience, but it may also impact the dataset's realness. We evaluated RNN performance using only a limited part of the PPMI and missed other input diagnosis features such as biospecimens, genes, and MRI/SPECT documents. Properly managing missing data can minimize data loss. Therefore, we excluded visits from the PPMI database with missing medication logs and severity scores to better understand missing values.

Finally, to explore the potential correlation between previous and subsequent doses, we calculated Pearson’s correlation coefficient. There was a moderate, positive correlation between the previous and next visits' LEDD, which was statistically significant (r = 0.639, p < 0.001). Hence, as the dose increases from patient to patient in the previous visit, the subsequent visits tend to see an increase in dosage as well. This implies that past doses may aid in predicting future doses, but other factors also influence dosing decisions. This observation confirms the need to include additional clinical factors listed in Table 1 to improve dose predictions.

## Conclusion

This research aimed to accurately predict the medication dosage for Parkinson's disease patients using sequence-by-sequence time series recurrent neural networks. To test the effectiveness of this methodology, we evaluated the performance of various recurrent neural networks, including Simple-RNN, LSTM, and GRU, using the PPMI database. The study found that LSTM and GRU networks were able to predict the correct medication amount for each PD patient based on their previous visits with great accuracy. These networks use memory blocks to analyze important information from earlier visits to inform future treatments. To compare the effectiveness of these recurrent neural networks with other regression models, we also applied the MLP model with no recurrent connections and in-time input samples with no visit history. The MLP model with the same architecture failed to track changes and only produced a constant output close to the average of all visits’ dosages, regardless of input features. Ultimately, the research demonstrated that recurrent neural networks, specifically LSTM and GRU, were superior in predicting the correct medication dosage for PD patients compared to other regression models.

## Data Availability

The dataset generated and analyzed during the current study was extracted from the PPMI database. Up-to-date information is available at www.ppmi-info.org by request.
